# Information Quality, Content Scope, and Audience Engagement of Popular Turkish Instagram Posts on Tooth Whitening: A Cross-Sectional Content Analysis

**DOI:** 10.3390/healthcare14101376

**Published:** 2026-05-18

**Authors:** Nasibe Aycan Yılmaz, Burçin Aydemir Akkoçan, Oğuzhan Akkoçan, Sude Eylül Ulucan

**Affiliations:** 1 Department of Restorative Dentistry, Faculty of Dentistry, Dokuz Eylül University, Balçova 35340, Izmir, Turkey; burcin.aydemir@deu.edu.tr (B.A.A.); sudeeylul.ulucan@deu.edu.tr (S.E.U.); 2 Department of Dental Biomaterials, School of Health Sciences, Dokuz Eylül University, Balçova 35340, Izmir, Turkey; oguzhan.akkocan@gmail.com

**Keywords:** tooth whitening, social media, oral health information quality, misinformation, content analysis, engagement rate

## Abstract

**Highlights:**

**What are the main findings?**

- Dentist/clinic accounts demonstrated significantly higher information reliability scores than independent users and brand accounts (median Modified Treatment-Information Reliability score: 3 vs. 2; *p* < 0.001), whereas content scope did not differ significantly by source; DIY whitening posts, predominantly produced by independent users, exhibited substantially lower content scope scores than in-office and multi-method posts and among the lowest information reliability scores across all approach categories, indicating a safety information deficit in the most audience-dominant content category.
- Reel posts achieved significantly higher content scope and information reliability scores than photo posts (*p* < 0.001 and adj. *p* = 0.001, respectively) and were associated with higher normalized audience engagement within this algorithmically curated sample; because Engagement Rate served simultaneously as the sampling criterion and as the outcome variable, this association reflects the internal composition of high-visibility content under platform curation and does not support causal or platform-neutral inference.

**What are the implications of the main findings?**

- Independent users commanded substantially greater raw audience reach (median likes: 293 vs. 20 for dentist/clinic accounts) yet predominantly produced DIY-focused content of low reliability, creating a structural public health risk in which low-quality information disproportionately dominates high-visibility content streams; this category encompasses a heterogeneous mix of lay users and potentially undisclosed commercial influencers, whose distinct policy implications, for health literacy interventions versus advertising transparency regulation, warrant analytical separation in future research.
- Within this algorithmically curated sample, producing evidence-based content in Reel format was not associated with reduced normalized audience engagement, suggesting that professional quality and platform visibility may be compatible rather than competing objectives; this hypothesis-generating observation warrants confirmation in prospective studies employing platform-neutral sampling before informing content strategy recommendations for dental professionals and public health communicators.

**Abstract:**

**Background:** Social media platforms, particularly Instagram, have become significant sources of health information, yet the quality of dental content remains underexplored. This study compared the scope and reliability of information in popular Turkish-language Instagram posts on tooth whitening by poster source and examined associations with post format, purpose, and whitening approach. As a secondary aim, the association between information quality and normalized audience engagement was investigated within this algorithmically curated sample. **Methods:** This cross-sectional content analysis included 500 publicly accessible Turkish Instagram posts retrieved under the hashtag #dişbeyazlatma. The posts were classified by source, purpose, format, and whitening approach. Content scope and information reliability were assessed using the Descriptive Coverage Index (DCI) and Modified Treatment-Information Reliability (MTIR) scores by two calibrated evaluators. Engagement Rate was calculated as (likes + comments)/follower count × 100. **Results:** Most posts originated from dentist/clinic accounts (75.8%) and were marketing-oriented (72.0%). Dentist/clinic accounts demonstrated significantly higher MTIR scores than independent users and brand accounts (*p* < 0.001), whereas DCI did not differ significantly across sources. Raw engagement differences disappeared after normalization (*p* = 0.408). Reel posts scored higher than photo posts on both measures; carousel posts scored higher than photos on MTIR but not DCI. In-office whitening content scored significantly higher than DIY- or OTC-focused posts on both measures (*p* < 0.001). A weak positive association was observed between MTIR and Engagement Rate (r = 0.141). **Conclusions:** Popular Turkish Instagram posts on tooth whitening exhibited substantial variability in content scope and reliability. Independent users commanded greater raw audience reach yet predominantly produced DIY-focused content with substantially lower content scope scores than in-office and multi-method posts, and among the lowest reliability scores, raising a public health concern within this high-visibility content stratum. These findings may inform content development strategies for dental professionals and public health communicators targeting Turkish-speaking audiences.

## 1. Introduction

In recent years, rapid advances in the internet and other digital technologies have substantially changed how people access health-related information. Social media platforms have become major channels for sharing, distributing, and retrieving health information, allowing users to seek advice, exchange experiences, and communicate directly with health professionals [1,2,3]. In this context, platforms such as Instagram, Facebook, YouTube, and X (formerly Twitter) are increasingly used not only for health communication and patient education, but also for the public presentation and promotion of healthcare services [2,3,4]. As a result, social media now plays an important role in shaping how health information is encountered, interpreted, and used in everyday decision-making.

Dentistry is particularly visible on visually oriented social media platforms, especially Instagram, because treatment outcomes can often be directly observed and easily presented through images and videos [5]. Dentists and clinics use social media to showcase treatment outcomes, provide patient information, and increase professional visibility [3,6]. At the same time, a considerable proportion of publicly available dental content is not subject to scientific review and may include promotional messages that are ethically, legally, and professionally debatable [5]. Although social media can facilitate communication between dental professionals and patients, it can also disseminate inaccurate, incomplete, or commercially biased information [5]. For this reason, the quality and reliability of oral health information shared on social media are of increasing concern.

Oral health literacy broadly encompasses the capacity to obtain, process, and understand basic oral health information needed to make appropriate health decisions, a concept rooted in broader health literacy frameworks [7]. In digital environments, this capacity may be challenged by the rapid circulation of inaccurate, incomplete, or commercially driven messages that reach large audiences without adequate scientific scrutiny [4,5]. This challenge is especially relevant in esthetic dentistry, where strong visual appeal, high public interest, and the potential for self-directed practices may amplify the influence of misleading or incomplete information before professional consultation [8]. With the widespread use of social media, public awareness of and interest in esthetic dental procedures have been reported to increase markedly, as documented in Gulf-region populations [9,10]; whether equivalent trends characterize the Turkish population specifically warrants further investigation. In Turkey, 90.9% of individuals aged 16–74 used the internet in 2025, and Instagram was among the most widely used social media platforms, with a 65.4% usage rate among internet users. These figures indicate high digital penetration in Turkey and suggest that a substantial proportion of the population may encounter health-related content on these platforms, including when researching dental treatment options [11]. In particular, demand for esthetic procedures such as the “Hollywood smile,” laminate veneers, and tooth whitening has been observed alongside social media trends and posts shared by highly followed independent users [9]. This growing reliance on social media for treatment-related information underscores the importance of evaluating the quality and reliability of the content encountered by the public in digital environments.

Tooth whitening is one of the most conservative and commonly performed procedures in esthetic dentistry, used to reduce tooth discoloration and improve dental appearance [12]. Interest in tooth whitening has increased substantially alongside rising esthetic expectations and the widespread promotion of the ideal smile on social media [13]. Although tooth whitening procedures and product sales are subject to regulatory restrictions in Turkey, the relatively easy access to over-the-counter (OTC) products may encourage individuals to pursue whitening practices outside professional supervision [12,14]. In this context, economic considerations may further amplify the tendency toward self-directed whitening; professional in-office procedures represent a considerable financial outlay relative to OTC and DIY alternatives, potentially making lower-cost self-administered options more attractive to a price-sensitive segment of the Turkish population, irrespective of the associated safety limitations. At the same time, the growing popularity of whitening has led to an intense flow of content on social media generated not only by dentists, but also by commercial companies and independent users, much of which may be marketing-oriented, scientifically limited, or insufficiently verified [12,13]. Previous studies have shown that individuals frequently obtain oral and dental health information from social media sources, although a substantial proportion of this information may be incomplete or inaccurate [15,16]. In this respect, whitening-related content may represent an important source of misinformation, with the potential to influence treatment preferences and health-related decision-making in undesirable ways [8]. Such misinformation in social media environments makes it more difficult for patients to access accurate oral health information and creates new challenges for dental professionals in patient counseling and management. Therefore, evaluating the scientific reliability, informational value, and communication quality of tooth whitening content shared on social media is clearly important [15,16]. Although previous studies have assessed the quality of dental content on social media, most have focused on English-language material and international populations, with only a limited number addressing Turkish-language content specifically [12,13,17]. Recent cross-sectional analyses have extended this line of inquiry to non-English-language contexts; for instance, Li et al. (2026) evaluated the quality and reliability of periodontitis-related videos on Chinese social media platforms (TikTok and Xiaohongshu) using the Modified DISCERN tool and engagement metrics, and found that dental professionals produced higher-quality content than non-medical uploaders, with persistent gaps in overall reliability across both platforms [18]. Nonetheless, such studies have addressed distinct clinical topics, video-based content formats, and sociocultural and regulatory contexts that differ substantially from those of Turkey. By contrast, studies specifically examining Turkish-language content and posts directed toward the Turkish population remain limited [17], and to the authors’ knowledge, no prior study has investigated the quality or audience engagement of tooth-whitening-specific content on Instagram within a Turkish-language context, based on a search of PubMed, Scopus, and Web of Science conducted prior to data collection. Since social media content may vary substantially across linguistic, cultural, and regulatory contexts, evaluating posts shared under Turkish-language hashtags is important for assessing the quality of oral health information available to this population. 

Beyond individual-level quality concerns, the present findings raise questions about the institutional scaffolding within which digital dental health content circulates. Although professional authorship is consistently associated with higher content reliability, professional origin alone does not guarantee that content meets established standards for evidence-based health communication. The observation that 72.0% of sampled posts were marketing-oriented, including a substantial proportion from dentist/clinic accounts, illustrates that a licensed professional source is a necessary but not sufficient condition for content quality. The World Health Organization has increasingly emphasized the role of healthcare institutions in curating and endorsing digital health content as a complement to individual practitioner communication [19]. In the absence of such institutional quality standards, professionally authored content remains structurally vulnerable to commercial bias, a concern directly relevant to the regulatory context described in Section Legal and Ethical Considerations. Furthermore, misinformation regarding DIY whitening techniques should not be characterized merely as an information gap: it constitutes a patient safety concern with concrete clinical implications, including enamel erosion, mucosal irritation, and use of hydrogen peroxide concentrations exceeding regulatory thresholds [8].

The paradigm of health communication is undergoing a significant shift from a traditional top-down information-delivery model to a digital era of supervision. In this new landscape, health literacy is no longer defined solely by an individual’s ability to find and understand information, but rather by the quality of the digital ecosystem and the presence of institutional content curation. As the volume of user-generated content on social media escalates, the role of professional dental organizations as “digital gatekeepers” becomes critical to ensure that e-health literacy is supported by evidence-based, verified resources [20]. Whether tooth whitening content on a high-penetration platform such as Instagram meets this standard remains empirically unexamined in the Turkish-language context.

Accordingly, the present study aimed to compare the scope and reliability of information in popular Turkish Instagram posts about tooth whitening by poster source. It also examined whether post format, posting purpose, and whitening approach were associated with these measures, and whether higher-quality information was related to normalized audience engagement. Two research questions were addressed: (1) whether posts from dentists or clinics demonstrated greater information reliability than posts from independent users or brand accounts (primary aim); and (2) as a secondary, exploratory question, whether information quality was associated with normalized audience engagement within this algorithmically curated sample, an association that should be interpreted with caution given that engagement also served as an implicit inclusion criterion for Top Posts.

## 2. Materials and Methods

### 2.1. Study Design

This study was designed as a cross-sectional, observational, descriptive content analysis to evaluate the content scope and information quality of Turkish-language Instagram posts related to tooth whitening. This study was reported in accordance with the Strengthening the Reporting of Observational Studies in Epidemiology (STROBE) guidelines for cross-sectional studies [21]; the completed STROBE checklist is provided as Supplementary Table S4.

Ethical review and approval were not required for this study under the applicable national regulatory framework. The study analyzed exclusively publicly accessible, anonymized social media content and did not involve human participants, the collection of identifiable personal data, or direct interaction with users. Under Turkish national regulations, including the Personal Data Protection Law (Law No. 6698, Article 5/2(d)) and the official ethics policy of TÜBİTAK ULAKBİM TR Dizin, secondary analyses of publicly accessible, anonymized digital content that involve no participant interaction or identifiable data do not require ethics committee review, as was the case in similar studies [16,18]. Throughout the study, principles of privacy and anonymity were strictly maintained; no information that could identify participants was collected, analyzed, or reported.

### 2.2. Data Source and Sample Selection

To reduce the potential influence of algorithmic personalization bias on sample selection, a standardized data collection approach was adopted [4,6], including the creation of a dedicated new account with no prior search history, user interactions, or follow relationships. Location services were disabled, in-app recommendations were restricted, and no users or content were followed throughout the data collection period. Data were collected using an iPhone 14 (Apple Inc., Cupertino, CA, USA; iOS 18.1) and the Instagram application version 305.0.

Data collection was completed between 1 and 5 November 2025 to minimize the effects of short-term algorithmic fluctuations. Following a preliminary screening of commonly used Turkish whitening-related hashtags, #dişbeyazlatma was selected because it ranked among the Turkish-language hashtags with the highest number of publicly accessible, relevant posts (approximately 316,000 at the time of screening). Posts listed in the “Top Posts” section under this hashtag were screened, after applying predefined inclusion and exclusion criteria; 500 posts were included in the final analysis. Because no established parameter exists for formal a priori sample size calculation in hashtag-based Instagram content analyses using Top Posts sampling, the sample size was determined pragmatically based on feasibility and consistency with the upper range of comparable dental social media studies [3,6,12]. A formal power calculation was not feasible due to the absence of normative parameters for this content analysis design, a limitation of the study. It is noted that post hoc observed power estimation is not recommended as a basis for sample adequacy inference, as it is mathematically redundant with the *p*-value and does not provide independent information about study power [22]. Additionally, the present study did not formally assess thematic saturation, that is, whether posts sampled beyond approximately *n* = 400 continued to yield substantively new content categories relative to those already identified. Future content analyses employing hashtag-based sampling should consider reporting the point at which incremental sampling produces diminishing thematic returns as a complementary indicator of sample adequacy alongside pragmatic feasibility criteria. The large effect sizes observed (e.g., η^2^ = 0.348 for whitening approach × Descriptive Coverage Index) are reported solely as descriptive magnitude indicators, not as retrospective power justifications [23]. The use of “Top Posts”, a section curated by Instagram’s engagement-based algorithm, was selected to capture content with high algorithmic visibility, reflecting the material most likely to be encountered during hashtag-based browsing [6]. This sampling strategy introduces two related limitations. First, the algorithm may systematically overrepresent content with rapid engagement velocity, potentially at the expense of scientific rigor, a pattern documented across healthcare social media contexts, including oncology and vaccine communication [24,25]. Second, because engagement metrics determined both inclusion in the sampling frame and served as an outcome variable in the present analysis, a degree of selection-on-outcome circularity should be considered when interpreting engagement-related findings [18].

**Figure 1:**
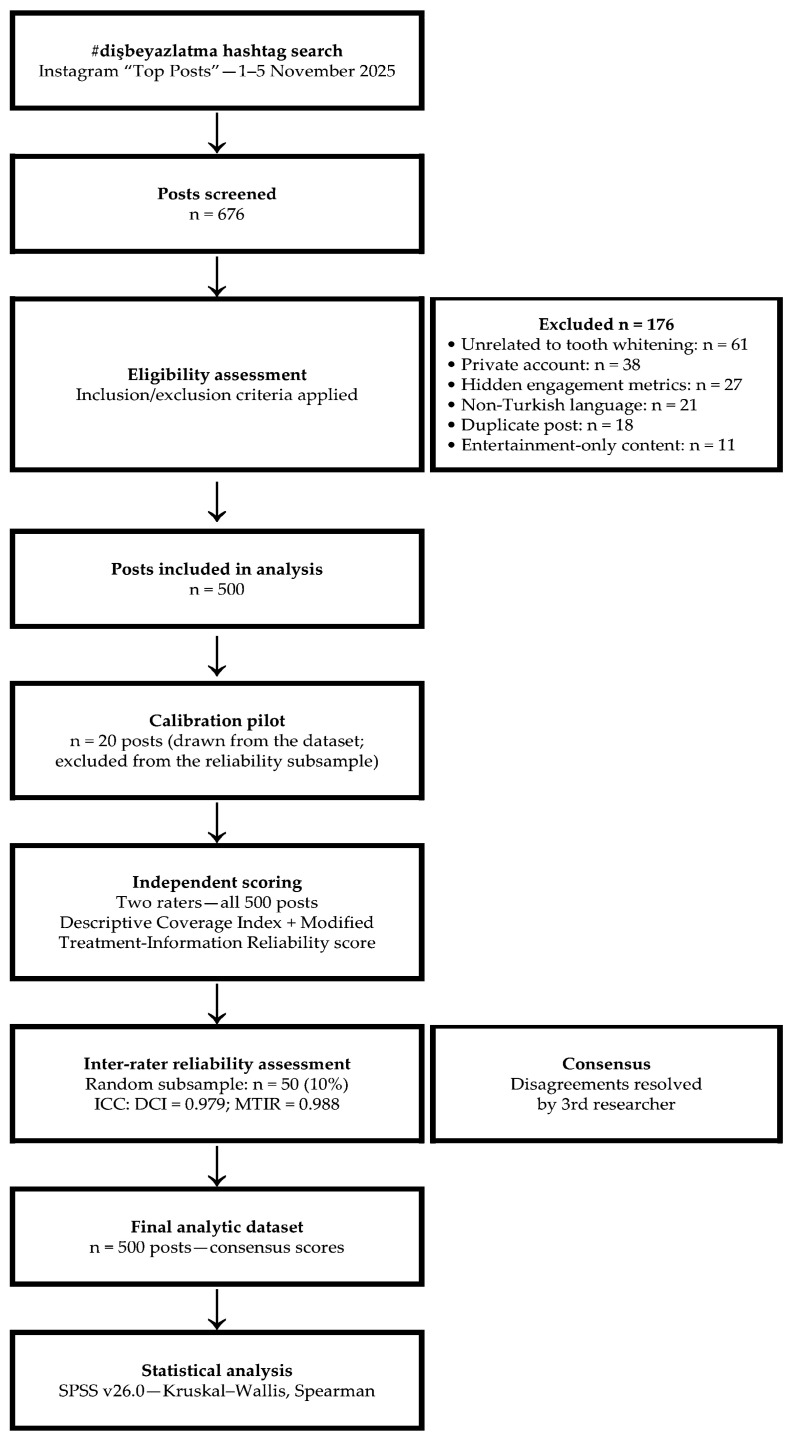
Study flow diagram for selecting Instagram posts on tooth whitening (#dişbeyazlatma). ICC = intraclass correlation coefficient; DCI = Descriptive Coverage Index; MTIR = Modified Treatment-Information Reliability score.

Posts were included if they were directly related to tooth whitening (vital teeth only), written in Turkish, and shared on public profiles with fully accessible content metrics. Exclusion criteria comprised: unrelated content, non-vital tooth whitening procedures, posts from private accounts, hidden engagement metrics, non-Turkish language, duplicate posts, and entertainment-only content (operationally defined in the decision rulebook; Supplementary Table S3). Of 676 screened posts, 176 were excluded (no direct relevance to tooth whitening: *n* = 61; private account: *n* = 38; hidden metrics: *n* = 27; non-Turkish: *n* = 21; duplicate: *n* = 18; entertainment-only: *n* = 11). The final analytic sample comprised 500 posts (Figure 1).

### 2.3. Data Recording and Classification

A structured data recording protocol adapted from the recent literature was developed for systematic analysis [6]. Each post was classified according to: (1) source of the post (dentist/clinic accounts, independent users, or brand/commercial accounts), based on publicly visible profile characteristics; (2) posting purpose (educational, marketing, or experience-based), determined by the dominant content intent, using a pre-established decision rulebook (Supplementary Table S3); (3) post format (photo, Reel, or carousel); and (4) whitening approach (in-office, OTC, DIY, multiple methods, or unspecified), also defined in Supplementary Table S3. Clinician-supervised take-home whitening trays were classified under the in-office whitening category on the grounds that the defining clinical characteristics of this approach, professional examination, custom tray fabrication, and dispensing under dental supervision, occur within the clinical setting, even though the whitening agent is subsequently applied by the patient at home. This classification decision was specified a priori in the decision rulebook (Supplementary Table S3) and applied consistently throughout. In cases where the account type or posting purpose was ambiguous, classification was determined by consensus between the two evaluators; where uncertainty persisted, a third senior researcher was consulted [6,16].

Follower, like, and comment counts were recorded as numerical variables. To reduce follower-size bias, an Engagement Rate was calculated for each post as: (likes + comments)/follower count × 100, following the approach used in comparable dental social media studies [6,12]. The follower, like, and comment counts were recorded at the time of archiving; these values represent a temporally fixed snapshot and may not reflect counts at the time of original posting [12,13]. All the sample accounts had at least six followers (minimum: 6), precluding undefined Engagement Rate values.

### 2.4. Content Evaluation

The posts were evaluated using the Descriptive Coverage Index (DCI; maximum possible: 8 points; observed range: 0–7), a descriptive information-coverage instrument adapted from Buldur et al. (2023) [12], and the Modified Treatment-Information Reliability score (treatment-information reliability; maximum 8 points). Both instruments have been used in previous dental social media content analyses [12,13]. The DCI assessed the scope of clinically relevant information presented about tooth whitening. The original instrument employed a binary scoring system from the outset, awarding 1 point for each of the 8 content parameters present in a post (maximum score: 8), with no Likert-type rating involved. The present study, therefore, adopted the DCI without any modification to its scoring format.

Treatment-information reliability was assessed using an adaptation of Section 2 and Section 3 of the original DISCERN instrument [26]; Section 1, which assesses source citation, currency, and aims disclosure, was excluded because it is structurally incompatible with short-form social media content [12]. This operationalization is referred to throughout as the Modified Treatment-Information Reliability score to reflect the structural adaptations applied and to distinguish it from the full original instrument. The complete scoring criteria for both instruments are provided in Supplementary Tables S1 and S2; item-level response frequencies are reported in Supplementary Tables S6 and S7. It should be noted that the Descriptive Coverage Index has been employed in a limited number of dental social media studies and has not been independently validated beyond its original application. Specifically, no published evidence of construct validity, criterion validity, or cross-study measurement equivalence exists for this instrument. While the excellent inter-rater reliability achieved in the present study (ICC = 0.979) demonstrates coding consistency, internal reliability does not substitute for construct validity; the instrument may systematically include or exclude content domains that do not fully capture clinically meaningful scope. This absence of external validation reflects a broader methodological limitation of the field, in which content-analytic instruments are frequently adapted from prior studies without undergoing formal psychometric evaluation. Accordingly, the DCI is treated as a descriptive information-coverage index in the present study. Future research should prioritize the systematic psychometric evaluation of this instrument within the specific context of cosmetic dentistry; concretely, this would involve establishing criterion validity by comparing DCI ratings against expert panel assessments, assessing measurement equivalence across social media formats and languages, and evaluating responsiveness to clinically meaningful differences in whitening content quality. Such a validation program would provide the field with a reproducible, evidence-based tool for cross-study comparison of digital dental health content.

A binary scoring system was used for both instruments: 1 if the criterion was present, 0 if absent. This approach was preferred over graded scoring to reduce interpretive ambiguity in short-form content, where the visual and textual brevity of posts limits the extent to which a criterion’s partial presence can be meaningfully distinguished from its full presence. Graded scoring across 8 items could, in principle, yield more fine-grained distributional properties and more consistent psychometric behavior; however, given the absence of validated ordinal anchors for these criteria in short-form social media contexts, selecting a binary approach was a more defensible, consistency-preserving choice. The scoring format was not modified from that used in the source instrument [12], and this methodological decision constitutes a limitation that should be considered when comparing score distributions across studies using graded alternatives. For comparative reporting, both scores were grouped into three descriptive categories (low: 0–2; moderate: 3–5; high: ≥6) following cutoffs applied in previous studies [12,13,27]. These categories were used solely to facilitate cross-measure descriptive comparison and should not be interpreted as clinically validated thresholds.

### 2.5. Calibration and Inter-Rater Reliability

Before the formal evaluation phase, the two assessors jointly reviewed a pilot subset of 20 posts to harmonize the interpretation of coding categories and scoring criteria [6]. These pilot posts were selected from the dataset but were not included in the reliability subsample; the pilot phase was used exclusively for calibration. All 500 posts were then independently scored by both evaluators. Inter-rater agreement was assessed in a randomly selected subsample of 50 posts (10% of the total sample) using the intraclass correlation coefficient (ICC) with a two-way mixed-effects absolute-agreement model. ICC values were calculated from independent ratings prior to any consensus resolution. Disagreements identified across the full 500-post evaluation were resolved through a consensus in discussions moderated by a third senior researcher, and final scores were assigned based on the agreed ratings. The ICC for the Descriptive Coverage Index was 0.979 (95% CI: 0.963–0.988; *p* < 0.001) and for the Modified Treatment-Information Reliability score was 0.988 (95% CI: 0.979–0.993; *p* < 0.001), indicating excellent agreement [28].

### 2.6. Statistical Analysis 

Statistical analysis was performed using IBM SPSS Statistics (Version 26.0; IBM Corp., Armonk, NY, USA). Descriptive statistics were presented as medians and interquartile ranges (IQRs). All continuous variables deviated significantly from normality (Kolmogorov–Smirnov and Shapiro–Wilk tests; all *p* < 0.001), supporting the use of non-parametric methods throughout. For comparisons across more than two independent groups, the Kruskal–Wallis test was applied with eta-squared (η^2^) as the effect size measure, calculated as η^2^ = H/(N − 1) [29]. Bonferroni-adjusted post hoc pairwise comparisons were performed when significant differences were detected. Associations between categorical variables were evaluated using Pearson’s chi-square test (with Cramér’s V as the effect size) or the Fisher–Freeman–Halton exact test when expected cell counts were insufficient to meet asymptotic assumptions. Relationships between continuous variables were examined using Spearman’s correlation analysis, including associations between the two scoring instruments (Descriptive Coverage Index and Modified Treatment-Information Reliability score) and between each instrument and Engagement Rate. Engagement Rate was also compared across quality-score categories as a secondary outcome. A two-sided *p*-value < 0.05 was considered statistically significant. To assess whether the source-level difference in the Modified Treatment-Information Reliability score persisted after accounting for the whitening approach, two supplementary analyses were conducted. First, an ordinal logistic regression was performed with Modified Treatment-Information Reliability score category (low/moderate/high) as the outcome, poster source as the primary independent variable, and whitening approach as a covariate (logit link; proportional odds model). Second, approach-stratified Kruskal–Wallis tests were conducted, with Mann–Whitney U tests applied when only two source groups were represented, to compare Modified Treatment-Information Reliability and Descriptive Coverage Index across source categories within each whitening approach stratum. In the ordinal logistic regression, brand/commercial accounts were specified as the reference category because they constituted the smallest and most homogeneous poster source group, providing a stable baseline for comparison. The results of these supplementary analyses are presented in Supplementary Table S5. Because multiple statistical comparisons were conducted across several independent variables, a Bonferroni correction was applied within each set of post hoc pairwise comparisons following significant Kruskal–Wallis tests. No global correction was applied across the full set of primary comparisons, as each addressed a distinct analytical domain, and the design was exploratory rather than confirmatory. The Engagement Rate variable showed marked right skew (mean = 5.16, SD = 16.13; range 0.00–222.74). Accordingly, the statistically significant but weak Spearman correlation between Engagement Rate and Modified Treatment-Information Reliability score (r = 0.141) should be interpreted with caution. This association accounts for approximately 2% of the shared variance, though this is a rough approximation, as r^2^ is strictly applicable to Pearson correlations. This value is therefore provided only as an indicative measure of effect size and likely reflects the influence of sample size on statistical significance rather than a strong effect.

## 3. Results

### 3.1. Descriptive Statistics and Distribution Assessment

Descriptive statistics for the 500 Instagram posts are presented in Table 1. Most posts were shared by dentist/clinic accounts (75.8%; *n* = 379), followed by independent users (14.4%; *n* = 72) and brand accounts (9.8%; *n* = 49). Regarding the posting purpose, most posts were marketing-oriented (72.0%; *n* = 360), followed by educational (26.0%; *n* = 130) and experience-based (2.0%; *n* = 10) posts (Table 1). The mean number of followers was 38,735.25 ± 181,121.24; the mean number of likes per post was 798.46 ± 5005.11; and the mean number of comments per post was 11.65 ± 62.43. All continuous variables deviated significantly from normality (all *p* < 0.001); non-parametric methods and medians with IQRs were used throughout.

### 3.2. Content and Information Quality Scores

The mean Descriptive Coverage Index was 3.91 ± 1.41 (median: 4.00; range: 0–7). Most posts were classified as moderate quality (68.0%; *n* = 340), with 17.0% (*n* = 85) rated low and 15.0% (*n* = 75) rated high (Table 2). The mean Modified Treatment-Information Reliability score was 3.15 ± 1.52 (median: 3.00; range: 0–8). In terms of reliability categories, 51.4% (*n* = 257) were moderate, 40.0% (*n* = 200) were low, and 8.6% (*n* = 43) were high (Table 2). A significant positive correlation was observed between the Descriptive Coverage Index and the Modified Treatment-Information Reliability score (Spearman r = 0.629; *p* < 0.001).

### 3.3. Comparisons According to Source of the Post

No significant differences were found among source categories for the Descriptive Coverage Index (H(2) = 0.102; *p* = 0.950). In contrast, a significant difference was observed in the Modified Treatment-Information Reliability score (H(2) = 35.977; *p* < 0.001; η^2^ = 0.072). The dentist/clinic accounts had significantly higher Modified Treatment-Information Reliability scores than both the independent-user accounts and brand accounts (both *p* < 0.001; Table 3). However, poster source and whitening approach were strongly associated (Cramér’s V = 0.701), with dentist/clinic accounts primarily focusing on in-office whitening (which was associated with higher quality scores), whereas independent users more often presented DIY methods. Therefore, source-level differences in the Modified Treatment-Information Reliability score should be interpreted with this potential confounding in mind.

Two supplementary analyses were conducted to evaluate whether the source-level advantage in the Modified Treatment-Information Reliability score was independent of the whitening approach (Supplementary Table S5). In the ordinal logistic regression model, the proportional odds assumption was formally met (Test of Parallel Lines: χ^2^(6) = 9.449, *p* = 0.150). The overall model provided a statistically significant improvement over the null model (χ^2^(6) = 132.178, *p* < 0.001; Nagelkerke R^2^ = 0.276). After controlling for whitening approach, the dentist/clinic accounts were significantly more likely to achieve a higher Modified Treatment-Information Reliability score category than brand/commercial accounts (B = 1.265, Wald = 6.417, *p* = 0.011, OR = 3.54, 95% CI: 1.33–9.42), while the difference between the independent users and brand/commercial accounts did not reach statistical significance (B = 0.699, *p* = 0.106). Analyses stratified by approach provided further clarification. Within the in-office whitening stratum (*n* = 222), source-related differences in Modified Treatment-Information Reliability score did not reach statistical significance (H(2) = 5.675, *p* = 0.059). This finding should be interpreted in light of the severely limited group sizes for independent users and brand accounts. Approach-stratified analyses provided partial clarification. Within the OTC stratum (*n* = 85), the dentist/clinic accounts demonstrated significantly higher MTIR scores than brand/commercial accounts (adj. *p* = 0.022). Within the DIY stratum (*n* = 38), dentist/clinic accounts scored higher on both measures (MTIR: exact *p* = 0.003; DCI: exact *p* = 0.045), though the dentist/clinic cell (*n* = 5) is insufficient for reliable inference, and this result should be treated as directional only. The remaining strata were not interpretable owing to severely unequal group sizes. The full results are presented in Supplementary Table S5. Taken together, these exploratory analyses suggest that the reliability advantage of dentist/clinic accounts may not be solely attributable to their predominant focus on in-office whitening; however, sparse cells, marginal model fit, and severely unequal group sizes within approach strata mean that this observation should be treated as hypothesis-generating rather than confirmatory [18]; the marginal Deviance goodness-of-fit statistic (*p* = 0.043) further implies that the regression estimates should be treated as directional rather than quantitatively precise.

Analysis of raw engagement metrics showed that independent user accounts had significantly higher follower counts, likes, and comments than both dentist/clinic and brand accounts (all *p* < 0.001; η^2^ ranging 0.152–0.243). However, no significant difference was found between source categories for Engagement Rate (H(2) = 1.795; *p* = 0.408; Table 3).

### 3.4. Comparisons According to Post Format

Significant differences across post formats were observed for both the Descriptive Coverage Index (H(2) = 28.828; *p* < 0.001; η^2^ = 0.058) and the Modified Treatment-Information Reliability score (H(2) = 18.006; *p* < 0.001; η^2^ = 0.036). The Reel posts scored significantly higher than the photo posts on both measures: Descriptive Coverage Index (adj *p* < 0.001) and Modified Treatment-Information Reliability score (adj. *p* = 0.001). The carousel posts scored significantly higher than the photo posts in the Modified Treatment-Information Reliability score (adj. *p* = 0.006), but this difference did not reach statistical significance in the Descriptive Coverage Index after Bonferroni correction (adj. *p* = 0.073). Engagement Rates also differed significantly by post format (H(2) = 16.332; *p* < 0.001; η^2^ = 0.033). The Reel posts (median: 1.68; IQR: 4.29) had significantly higher Engagement Rates than the photo posts (median: 0.74; IQR: 2.43) (adj. *p* < 0.001), whereas the carousel posts (median: 1.50; IQR: 2.38) did not differ significantly from either the photo or Reel posts. The results are presented in Table 4.

### 3.5. Comparisons According to Whitening Approach

Statistically significant differences were observed across the whitening approach groups for both the Descriptive Coverage Index (H(4) = 173.791; *p* < 0.001; η^2^ = 0.348) and the Modified Treatment-Information Reliability score (H(4) = 128.808; *p* < 0.001; η^2^ = 0.258). The posts focused on in-office whitening and those discussing multiple methods had significantly higher scores on both measures than the posts focused on DIY methods or OTC products (all *p* < 0.001; Table 4).

### 3.6. Associations Between Categorical Variables

A strong, statistically significant association was found between poster source and whitening approach (χ^2^(8) = 491.404; *p* < 0.001; Cramér’s V = 0.701; Table 5). Among the dentist/clinic posts, the majority focused on in-office whitening (55.9%), whereas independent users’ posts primarily focused on DIY methods (45.8%) and OTC products (43.1%). Poster source was also significantly associated with posting purpose, as confirmed by the Fisher–Freeman–Halton exact test (exact *p* < 0.001). The dentist and clinic posts were more often educational or promotional; the independent-user posts were more often experience-based; and the brand accounts were predominantly promotional. Poster source was significantly associated with post format (χ^2^(4) = 85.129, *p* < 0.001; Cramér’s V = 0.292; Table 5). This association was confirmed by Fisher–Freeman–Halton exact test (exact *p* < 0.001).

### 3.7. Engagement Levels According to Posting Purpose

When raw engagement metrics were compared by posting purpose, the experience-based posts had substantially higher like and comment counts than the educational and marketing posts (likes: H(2) = 27.307; *p* < 0.001; η^2^ = 0.055; comments: H(2) = 15.724; *p* < 0.001; η^2^ = 0.032; Table 4). These results should be interpreted with particular caution, as the experience-based category comprised only 10 posts (2.0% of the analytic sample), and the statistical significance of the omnibus test is likely driven by a small number of high-engagement outliers rather than reflecting a stable distributional pattern. Importantly, the normalized Engagement Rates did not differ significantly by posting purpose (H(2) = 0.575; *p* = 0.750; η^2^ < 0.001), as reported in the Table 4 footnote.

### 3.8. Relationship Between Information Quality and Engagement Rate

Engagement Rates were compared across quality-score categories. No significant difference was found across Descriptive Coverage Index categories (H(2) = 3.977; *p* = 0.137). In contrast, a significant difference was detected among Modified Treatment-Information Reliability score categories (H(2) = 8.842; *p* = 0.012).

Post hoc analysis showed that within this engagement-based sampling frame, posts with high Modified Treatment-Information Reliability scores (median: 1.85; IQR: 4.61) achieved significantly higher Engagement Rates than posts with low scores (median: 0.88; IQR: 2.55) (adj. *p* = 0.033; Table 6). However, this finding must be interpreted in light of a structural circularity inherent to the study design: Engagement Rate served simultaneously as the criterion for inclusion in the Top Posts sampling frame and as the outcome variable in the present analysis. Consequently, the direction and magnitude of the quality–engagement relationship cannot be established with certainty, and the observed pattern should not be extrapolated to predict how information quality might affect engagement under platform-neutral sampling conditions. Spearman’s correlation analysis confirmed a statistically significant but weak positive relationship between Modified Treatment-Information Reliability scores and Engagement Rate (r = 0.141; *p* = 0.002), accounting for approximately 2% of the variance in Engagement Rate (a rough approximation based on r^2^, provided as a descriptive indicator only). However, the relationship between Descriptive Coverage Index and Engagement Rate was not significant (r = 0.061; *p* = 0.173; Table 5). Within this sampling frame, higher reliability was not associated with lower normalized engagement. The observed pattern likely reflects the internal structure of high-visibility posts rather than a generalizable inference about the broader content ecosystem.

## 4. Discussion

The internet and social media have transformed health communication from a traditional one-way model into a dynamic ecosystem in which patients can access information instantly and shape their treatment-related decisions accordingly [1,2]. In esthetic dentistry, where visual outcomes play a particularly prominent role, patients show a strong tendency to review independent users’ experiences, dentists’ portfolios, and before-and-after images related to topics such as the “Hollywood smile” and tooth whitening, before presenting for clinical care [6]. Within this high-volume information environment, hashtags serve as key tools for categorizing content and enhancing its visibility to target audiences [1,6]. In this context, the hashtag #dişbeyazlatma was selected for analysis because it was among the Turkish-language hashtags with the highest number of relevant posts. It should be acknowledged, however, that the Top Posts section reflects Instagram’s engagement-based curation rather than a neutral sample of all available content; accordingly, engagement-related findings in this study are conditional on this pre-selection, as detailed in Section 3.8. This tendency to prioritize high-engagement content, potentially at the expense of scientific rigor, has been documented in other healthcare social media contexts, including oncology and vaccine communication [24,25].

The findings of the present study indicate that popular Turkish Instagram content on tooth whitening is heterogeneous in terms of informational scope and reliability. Although the informational scope did not differ significantly by poster source, dentist/clinic accounts demonstrated higher Modified Treatment-Information Reliability scores than independent users and brand accounts, suggesting that professional authorship may be associated with more reliable digital oral health communication. The moderate-to-strong positive correlation between Descriptive Coverage Index and Modified Treatment-Information Reliability score (r = 0.629) further suggests that posts covering a broader range of whitening-related topics may also be more likely to present information in a more balanced and trustworthy manner. Nevertheless, these two instruments assess related but distinct dimensions of information quality, and their differing patterns across source categories support their use as complementary rather than redundant measures.

The most important interpretive constraint on source-level comparisons in this dataset is the near-complete collinearity between poster source and whitening approach (Cramér’s V = 0.701), a central structural finding rather than a mere nuisance covariate. Because dentist/clinic accounts primarily focused on in-office whitening, which was associated with substantially higher quality scores, and independent users predominantly focused on DIY methods, which were associated with lower scores, the source-level difference in Modified Treatment-Information Reliability score is substantially confounded by content domain. To address this directly, two supplementary analyses were conducted. The ordinal logistic regression model satisfied the proportional odds assumption (Test of Parallel Lines: *p* = 0.150). However, the Deviance goodness-of-fit statistic indicated marginal model misfit (*p* = 0.043), likely reflecting sparse cells arising from the strong association between poster source and whitening approach. This misfit implies that the point estimates of the odds ratios, including OR = 3.54 for dentist/clinic accounts, should be treated as approximate rather than precise, and the regression findings should be interpreted accordingly as directional rather than quantitatively definitive. With this caveat noted, the model confirmed that the dentist/clinic accounts were significantly more likely to achieve a higher Modified Treatment-Information Reliability score category than the brand/commercial accounts after controlling for whitening approach (OR = 3.54, 95% CI: 1.33–9.42, *p* = 0.011; Nagelkerke R^2^ = 0.276). Approach-stratified Kruskal–Wallis analyses further demonstrated that significant source-related differences in Modified Treatment-Information Reliability score were present within the OTC stratum (*p* = 0.028; dentist/clinic > brand, adj. *p* = 0.022). Within the DIY stratum (*n* = 38), the Mann–Whitney U test yielded a statistically significant result (exact *p* = 0.003); however, the dentist/clinic cell comprised only five posts, which is insufficient to support substantive interpretation. This result should therefore be regarded as consistent in direction with the overall source effect, but not as independent corroborating evidence. Taken together, while content domain substantially shapes quality scores, these analyses suggest that account type may contribute to information reliability beyond content domain, though this inference should be treated as directional given the sparse cells and marginal model fit noted above. This pattern is consistent with the findings reported by Li et al. (2026), who evaluated periodontitis-related short videos on TikTok and Xiaohongshu using the Modified Treatment-Information Reliability tool, and similarly found that dental professionals produced higher-quality content than non-medical uploaders with marked source-related differences in reliability scores [18]. This convergence supports the hypothesis that professional authorship contributes to information reliability across dental social media contexts, a pattern consistent with recent analyses of orthodontic content [31] and with earlier systematic evidence that health content quality varies substantially by source type [2]. Nevertheless, replication across additional platforms, clinical topics, and linguistic contexts is needed before broad generalization is warranted. These findings also underscore the importance of controlling for content domain when interpreting source-level comparisons, given the strong confounding between poster source and whitening approach observed in the present study [12,13].

A different pattern emerged for audience engagement on Instagram. Independent-user posts generated higher raw counts of likes and comments than posts shared by dentist/clinic or brand accounts; however, these differences were no longer evident after normalization for follower count, suggesting that the apparent engagement advantage largely reflects account size rather than greater per-follower interaction. Normalized engagement differed across Modified Treatment-Information Reliability quality categories (Kruskal–Wallis test significant); however, the underlying association was weak (Spearman r = 0.141; r^2^ ≈ 0.02). Given the pronounced right skew of Engagement Rate and the small number of high-quality posts (*n* = 43), this pattern is parsimoniously interpreted as evidence that higher information reliability was not associated with reduced engagement, rather than as evidence of a meaningful positive effect.

Post format and posting purpose provided further context for these findings. The Reel posts achieved higher scores on both content scope and information reliability than the photo posts; the carousel posts achieved higher reliability scores than the photo posts (adj. *p* = 0.006) and, notably, demonstrated a higher average rank than the Reel posts on this measure (291.38 vs. 271.53), though this difference did not reach statistical significance (adj. *p* = 1.000); the difference in content scope between the carousel and photo posts did not reach statistical significance after Bonferroni correction (adj. *p* = 0.073). These patterns are consistent with the greater content capacity of dynamic and multi-panel formats. This advantage may be particularly pronounced in dentistry, where clinically adequate communication frequently requires sequential presentation of procedural steps, safety precautions, contraindications, and expected outcomes, information that a single static image is structurally unable to accommodate. Reels afford temporal ordering of content, enabling creators to walk viewers through a whitening procedure step-by-step in a manner that maps naturally onto the logic of clinical explanation; carousel formats offer a comparable advantage through spatially ordered multi-panel disclosure. The higher reliability scores of these formats may therefore reflect not merely greater available space, but also a structural alignment between their affordances and the communicative demands of evidence-based dental health information. Regarding posting purpose, the experience-based posts attracted substantially greater raw engagement counts than the educational or marketing posts, a pattern consistent with the broader literature on audience responsiveness to personal narrative in digital health communication [2]. This observation must, however, be interpreted with caution given that the experience-based category comprised only 10 posts; the substantial raw engagement counts recorded for this group likely reflect a small number of high-reach outlier accounts rather than a systematic pattern generalizable to this content type. Nonetheless, these raw engagement differences did not persist after normalization for audience size (Engagement Rate: *p* = 0.750), suggesting that the apparent engagement advantage of experience-based posts primarily reflects account reach rather than superior interaction efficiency.

The engagement data add a strategically important dimension to this interpretation. The Reel posts demonstrated significantly higher normalized Engagement Rates than the photo posts (adj. *p* < 0.001), whereas the carousel posts did not differ significantly from either format. This pattern suggests that Reels distinctively combine informational capacity with audience reach in this sample, an alignment that carousel posts, despite their comparable reliability advantage over photos, did not replicate in terms of normalized engagement [6]. Carousel formats, while structurally capable of layered disclosure, do not appear to confer the same engagement advantage within this high-visibility sampling frame, which may reflect platform-level algorithmic prioritization of video content over static multi-panel posts. Whether this engagement pattern reflects genuine viewer preference for video-based dental content or algorithmic amplification of the Reels format cannot be determined from the present data and warrants direct investigation. In this regard, Li et al. (2026) reported that TikTok demonstrated significantly higher user engagement than Xiaohongshu for periodontitis-related videos, and that higher-quality content was not penalized in terms of audience interaction [18]; a convergent pattern was observed in the present study, in which the platform’s engagement-based curation did not appear to actively penalize evidence-informed content. As noted in Section 3.8, however, this observation is conditioned on the Top Posts sampling frame and cannot support causal inference; platform differences also preclude direct comparison with the Li et al. findings [18].

The observed reliability advantage of Reels and carousel posts, and the content scope advantage specific to Reels, should, however, be interpreted with an important caveat: poster source and post format were significantly associated (Cramér’s V = 0.292; Table 5), with the dentist/clinic accounts more frequently utilizing these formats. Consequently, the apparent format effect on information quality may partly reflect the higher reliability of professionally authored content rather than an independent contribution of format per se. Disentangling these contributions, whether it is the format that enables higher quality, the professional author who chooses the format, or both, was beyond the scope of the present study and represents a priority for future research employing designs capable of isolating format effects independently of source type.

Whether repeated exposure to highly visible social media content shapes patients’ prior expectations and treatment preferences before professional consultation was not assessed in this study; this question warrants direct investigation in future research, as it has direct implications for pre-consultation communication in esthetic dentistry [6,15]. These findings suggest that dental professionals may benefit from awareness of the whitening-related content their patients are likely to have encountered through highly visible social media posts [12,18]. Because of the cross-sectional observational design, the associations identified in this study should be interpreted as descriptive rather than causal.

### Legal and Ethical Considerations

The regulatory and ethical dimensions of social media dental advertising warrant explicit consideration in interpreting the present findings. In Turkey, medical and dental advertising is subject to restrictions under the Law on the Practice of Medicine and Medical Arts (Law No. 1219) and the professional ethical guidelines issued by the Turkish Medical Association, which limit promotional health communications by licensed practitioners. Additionally, tooth whitening products are regulated under the Turkish Cosmetic Regulation (Kozmetik Yönetmeliği, Annex III/12), which is harmonized with EU Directive 2011/84/EU and restricts the sale of products containing more than 0.1% hydrogen peroxide to dental practitioners [32,33]. The observation that 72.0% of sampled posts were classified as marketing-oriented raises the question of whether a substantial proportion of this content may operate at or beyond the boundaries of these regulatory frameworks, particularly where clinically licensed accounts engage in direct promotional communication. The present study was not designed to adjudicate regulatory compliance and did not apply legal criteria to post classification; accordingly, no inference regarding individual legal liability is made or implied. Nevertheless, the scale of marketing-oriented content in the highest-visibility stratum of a major dental health hashtag is a finding that may be of relevance to Turkish professional regulatory bodies and to researchers investigating the intersection of digital health communication and advertising law.

The present findings also speak to a broader transition in health communication: from a traditional one-way dissemination model toward what has been described as a “supervised digital health” ecosystem, in which authoritative institutions actively curate and endorse online content [19,20]. In the absence of such institutional scaffolding, the burden of evaluating dental information falls disproportionately on the patient, a burden that public e-health literacy interventions are specifically designed to address. E-health literacy, defined as the ability to seek, find, and critically appraise health information from electronic sources [34], has been shown to improve when official health bodies actively engage in content curation and digital health education [35]. Strengthening these institutional mechanisms represents a priority direction for dental public health communication, particularly in high-penetration social media environments such as Turkey’s [11].

Future studies should consider incorporating regulatory compliance assessment as a distinct analytical dimension, particularly in jurisdictions with explicit statutory frameworks governing health-related commercial communication on social media. More broadly, the present findings support the argument that professional authorship, while associated with higher reliability, is insufficient in the absence of institutional quality oversight: content produced by licensed professionals that is predominantly marketing-oriented may still fall short of the evidence-based communication standards recommended by WHO for digital health environments [19]. Strengthening public e-health literacy, defined as the ability to seek, find, and critically evaluate health information from digital sources [34,36], represents a complementary strategy, particularly for patients who lack the professional background to independently evaluate DIY whitening claims. The development and adoption of institutional endorsement mechanisms, such as professional society certification of social media dental health content, represents a priority direction for the field [19,37].

This study has several limitations. First, the analysis was restricted to Top Posts, which are shaped by Instagram’s platform-specific visibility algorithms and may not reflect the full range of whitening-related content. In addition, because engagement served both as a determinant of Top Posts inclusion and as an outcome under analysis, a degree of selection-on-outcome circularity should be acknowledged when interpreting the engagement-related findings. Second, the sampling frame was based on a single Turkish-language hashtag. It is also possible that #dişbeyazlatma overrepresents professionally generated content relative to other whitening-related hashtags, potentially biasing source-level comparisons toward higher reliability scores for dentist/clinic accounts. Third, the poster source was classified based on publicly visible account characteristics and could not be independently verified in all cases. Fourth, although the scoring framework was adapted from previously used instruments, the Descriptive Coverage Index was applied in its original binary format (1 point per criterion present; maximum 8 points). In addition, the exclusion of Section 1 from the original Modified DISCERN instrument also represents a deliberate structural adaptation. Neither instrument has been formally validated in Turkish-language short-form social media contexts. More broadly, the DCI, which is used here as a descriptive information-coverage index, lacks published evidence of external validation in any context: no study to date has established its construct validity, criterion validity, or measurement equivalence across samples. The Modified Treatment-Information Reliability score benefits from a stronger validation lineage through its derivation from the original Modified DISCERN instrument [26]; however, the structural adaptations applied here, binary scoring and the exclusion of Section 1, have not been independently evaluated. These absences represent a field-level limitation rather than a study-specific shortcoming, underscoring the need for systematic psychometric evaluation of content-analytic instruments used in dental social media research. In particular, future studies should develop and validate a content-analytic instrument specifically calibrated to the communicative and clinical demands of cosmetic dentistry social media content, including criterion validity assessment against expert consensus ratings and cross-platform measurement equivalence testing. Fifth, audience engagement should not be interpreted as a direct indicator of educational value or behavioral impact; higher visibility may reflect attention capture rather than knowledge acquisition [2]. Sixth, the present study did not assess whether the sampled content complied with Turkish regulatory restrictions on tooth whitening products and procedures. Seventh, the independent user category encompasses considerable internal heterogeneity, including DIY content creators, OTC product reviewers, and experience-based testimonial accounts, each with markedly different risk profiles and motivations. Grouping these accounts under a single category may therefore obscure meaningful within-group variation and should be considered when interpreting source-level comparisons. Of particular relevance, the present classification system did not distinguish between genuinely independent users and influencers who may have undisclosed commercial relationships with product brands. Where an account is nominally classified as “independent”, yet the predominant posting purpose is marketing-oriented, the reliability of the content may be structurally compromised in ways that the source classification alone does not capture. Future studies should incorporate disclosure status and commercial affiliation as explicit classification variables to address this ambiguity. Eighth, the supplementary ordinal logistic regression model showed marginal misfit on the Deviance goodness-of-fit statistic (*p* = 0.043), with the proportional odds assumption formally met (Test of Parallel Lines: χ^2^(6) = 9.449, *p* = 0.150); as discussed in Section 4, this misfit is attributable to sparse cells arising from the strong source–approach collinearity, and the OR estimates should be treated as directional rather than quantitatively precise. Future studies should employ oversampling of underrepresented source–content combinations to provide more precise point estimates. However, approach-stratified analyses were constrained by severely unequal group sizes across the multiple methods and by unspecified strata, which precluded meaningful comparisons within those categories. Finally, as data collection was completed within a five-day window in November 2025, the findings reflect a temporally fixed snapshot of a highly dynamic content environment [12,13].

## 5. Conclusions

Popular Turkish Instagram posts on tooth whitening showed substantial variability in both content scope and information reliability. Dentist/clinic accounts demonstrated higher reliability scores than independent users and brand accounts, yet commanded substantially lower raw audience reach; among the independent users, DIY-focused content, which was associated among the lower content scope scores across approach categories (median DCI = 3.50) and with information reliability scores among the lowest across all approach categories (median Modified Treatment-Information Reliability = 2.00, shared with OTC and unspecified/other groups), was the predominant whitening approach (45.8%). However, the content scope did not differ significantly by source. The source-level reliability difference should be interpreted in the context of strong confounding by the whitening approach. Post format and whitening approach were each significantly associated with content scope and information reliability; in-office-focused posts scored higher on both measures, Reels scored higher than photos on both measures, and carousel posts scored higher than photos on reliability but not on content scope. These associations should be interpreted with caution, however, as post format and whitening approach were both strongly associated with poster source, and no multivariate analysis was conducted to isolate their independent contributions. While independent users generated higher raw engagement metrics, these differences were no longer evident after normalization for audience size. Within this sampling frame, higher information reliability was not associated with reduced normalized audience engagement; this observation is hypothesis-generating and should not be interpreted as establishing a causal or platform-generalizable relationship, given the circularity inherent to the Top Posts sampling design (Section 3.8). Given the observational design and hashtag-based sampling frame, generalization beyond the conditions of this study is not warranted. Nonetheless, the descriptive patterns identified here may provide preliminary evidence to inform how dental professionals and public health communicators approach content development on social media platforms targeting Turkish-speaking audiences.

Future research should examine whether the source-level and format-related patterns identified here replicate across additional dental topics, platforms, and language contexts, and whether longitudinal or experimental designs can establish directional relationships between content quality and audience engagement that cannot be inferred from cross-sectional sampling. In particular, multivariable modeling approaches and stratified sampling designs with more balanced group sizes across poster source, post format, and whitening approach would better disentangle the independent contributions of each factor to information quality and engagement, addressing the near-complete collinearity constraint identified in the present study. Prospective studies examining how exposure to social media content of varying quality influences patient knowledge, treatment expectations, and clinical decision-making would further advance the evidence base for digital oral health communication.

## Figures and Tables

**Table 1:** The descriptive statistics and normality assessment of the included popular Turkish Instagram posts on tooth whitening.

Variable	*n*	%	Mean ± SD	Median (IQR)	Min–Max	K-S *p*/S-W *p*
Poster Source						
Dentist/Clinic	379	75.8	—	—	—	—
Independent User	72	14.4	—	—	—	—
Brand/Commercial	49	9.8	—	—	—	—
Post Format						
Photo	235	47.0	---			
Reels	217	43.4	---			
Carousel	48	9.6	---			
Posting Purpose						
Marketing	360	72.0	—	—	—	—
Educational	130	26.0	—	—	—	—
Experience-Based	10	2.0	—	—	—	—
Continuous Variables						
Follower Count	500	—	38,735 ± 181,121	2144 (8087)	6–2,400,000	<0.001/<0.001
Like Count	500	—	798 ± 5005	22 (46)	0–69,100	<0.001/<0.001
Comment Count	500	—	11.65 ± 62.43	0 (1)	0–887	<0.001/<0.001
Descriptive Coverage Index	500	—	3.91 ± 1.41	4.00 (2)	0–7	<0.001/<0.001
Modified Treatment-Information Reliability Score	500	—	3.15 ± 1.52	3.00 (2)	0–8	<0.001/<0.001

SD = standard deviation; IQR = interquartile range, reported as IQR width for all continuous variables; K-S = Kolmogorov–Smirnov; S-W = Shapiro–Wilk; —: not applicable for categorical variables. Non-parametric tests were used throughout owing to non-normal distributions. A median comment count of 0 for the dentist/clinic accounts indicates that most posts in this group received no comments. All the sample accounts had at least six followers (minimum: 6), precluding undefined Engagement Rate values. The medians and IQRs for the follower, like, and comment counts were derived from the full-sample SPSS EXAMINE output. Posting purpose categories are mutually exclusive; the percentages sum to 100%.

**Table 2:** Descriptive Coverage Index and Modified Treatment-Information Reliability score: descriptive and categorical distributions. (**A**) Continuous distribution, (**B**) categorical distribution.

(**A**)			
**Scale**	**Mean ± SD**	**Median (IQR)**	**Min–Max**
Descriptive Coverage Index	3.91 ± 1.41	4.00 (2)	0–7
Modified Treatment-Information Reliability Score	3.15 ± 1.52	3.00 (2)	0–8
(**B**)			
**Scale**	**Low, *n* (%)**	**Moderate, *n* (%)**	**High, *n* (%)**
Descriptive Coverage Index	85 (17.0)	340 (68.0)	75 (15.0)
Modified Treatment-Information Reliability Score	200 (40.0)	257 (51.4)	43 (8.6)

Low: 0–2 points; moderate: 3–5 points; high: ≥6 points. For comparative reporting only, the same numerical cutoffs were applied to both scales to facilitate descriptive comparison and do not imply conceptual equivalence between the two measures.

**Table 3:** Comparison of Descriptive Coverage Index, Modified Treatment-Information Reliability score, and engagement metrics by poster source.

Variable	Dentist/Clinic Median (IQR)	Independent User Median (IQR)	Brand/Commercial Median (IQR)	H (df)	*p*	η^2^
Descriptive Coverage Index	4.00 (2)	4.00 (2)	4.00 (2)	0.102 (2)	0.950	—
Modified Treatment-Information Reliability Score	3.00 (2)	2.00 (1)	2.00 (1)	35.977 (2)	<0.001	0.072
Engagement Rate (%)	1.27 (3.77)	1.22 (2.54)	0.56 (4.07)	1.795 (2)	0.408	—

Kruskal–Wallis test with Bonferroni-corrected post hoc pairwise comparisons where significant. Modified Treatment-Information Reliability score: dentist/clinic > independent user and brand/commercial (both *p* < 0.001). Engagement Rate did not differ significantly across source groups (*p* = 0.408). η^2^ = H/(N − 1). Raw engagement metrics (follower count, like count, comment count) differed significantly by source (all *p* < 0.001; η^2^ ranging from 0.152 to 0.243): independent users demonstrated substantially higher counts than dentist/clinic and brand/commercial accounts across all three measures. These comparisons are presented in the text (Section 3.3) rather than the table body, as they are secondary to the primary quality and engagement comparisons. Supplementary analyses addressing confounding by poster source and whitening approach are presented in Supplementary Table S5.

**Table 4:** Quality scores and engagement metrics by post format, whitening approach, and posting purpose. (**A**) Comparisons by post format, (**B**) comparisons by whitening approach, (**C**) comparisons by posting purpose.

(**A**)							
**Variable**	**Photo (*n* = 235) Median (IQR)**	**Reels (*n* = 217) Median (IQR)**	**Carousel (*n* = 48) Median (IQR)**	**H (df)**	***p***	**Post Hoc**	
Descriptive Coverage Index	3.00 (1)	4.00 (2)	4.00 (2)	28.828 (2)	<0.001	R > Ph	
Modified Treatment-Information Reliability Score	3.00 (1)	3.00 (2)	3.00 (3)	18.006 (2)	<0.001	R, C > Ph	
Engagement Rate (%)	0.74 (2.43)	1.68 (4.29)	1.50 (2.38)	16.332 (2)	<0.001	R > Ph	
(**B**)							
**Variable**	**In-Office MD (IQR)**	**Multi-Method MD (IQR)**	**OTC MD (IQR)**	**DIY MD (IQR)**	**H (df)**	***p***	**η^2^**
Descriptive Coverage Index	4.00 (2)	5.00 (2)	4.00 (2)	3.50 (1)	173.791 (4)	<0.001	0.348
Modified Treatment-Information Reliability Score	3.00 (2)	5.00 (2)	2.00 (1)	2.00 (2)	128.808 (4)	<0.001	0.258
(**C**)							
**Variable**	**Educational Median (IQR)**	**Marketing Median (IQR)**	**Experience-Based Median (IQR)**		**H (df)**	***p***	
Like Count	18 (34)	18 (42)	1199 (10,948)		27.307 (2)	<0.001	
Comment Count	0 (1)	0 (1)	24 (196)		15.724 (2)	<0.001	

Ph = photo; R = Reels; C = carousel. η^2^: Descriptive Coverage Index = 0.058; Modified Treatment-Information Reliability score = 0.036; Engagement Rate = 0.033. Post hoc (Descriptive Coverage Index): R > Ph (adj. *p* < 0.001); C vs. Ph: not significant (adj. *p* = 0.073). Post hoc (Modified Treatment-Information Reliability score): R > Ph (adj. *p* = 0.001), C > Ph (adj. *p* = 0.006); R vs. C: not significant (adj. *p* = 1.000). Notably, carousel posts had a higher average Modified Treatment-Information Reliability score than Reel posts (291.38 vs. 271.53), though the difference was not statistically significant. Post hoc (Engagement Rate): R > Ph (adj. *p* < 0.001); C vs. Ph and C vs. R: not significant. OTC = over-the-counter commercial products; DIY = do-it-yourself/natural home remedies. The unspecified/other group was included in the Kruskal–Wallis test (H(4); content: MD = 2.00, IQR = 1; Modified Treatment-Information Reliability: MD = 2.00, IQR = 1) but is omitted from the table body for conciseness. Post hoc: in-office and multi-method > OTC and DIY (*p* < 0.001 for both scores). Post hoc: experience-based > educational and marketing (*p* < 0.001 for like count and comment count). η^2^: like count = 0.055; comment count = 0.032. Engagement Rate did not differ significantly by posting purpose (H(2) = 0.575, *p* = 0.750); Engagement Rate medians: educational = 1.27, marketing = 1.22, experience-based = 1.35.

**Table 5:** Categorical associations and Spearman correlations. (**A**) Associations between categorical variables, (**B**) Spearman rank correlations.

(**A**)			
**Variables**	**χ^2^ (df)**	***p***	**Cramér’s V**
Poster source × Whitening approach	491.404 (8)	<0.001	0.701
Poster source × Posting purpose	— a	<0.001	—
Poster source × Post format	85.129 (4)	<0.001	0.292
(**B**)			
**Variable 1**	**Variable 2**	**Spearman r**	***p***
Descriptive Coverage Index	Modified Treatment-Information Reliability Score	0.629	<0.001
Engagement Rate	Modified Treatment-Information Reliability Score	0.141	0.002
Engagement Rate	Descriptive Coverage Index	0.061	0.173

a Poster source × posting purpose: Fisher–Freeman–Halton exact test was used as the primary analysis; therefore, the Pearson chi-square statistic is not reported. Poster source × post format: 1 cell (11.1%) had an expected count less than 5 (brand/commercial accounts × carousel: expected count = 4.70). Pearson’s chi-square test showed a significant association, χ^2^(4) = 85.129, *p* < 0.001, and Fisher–Freeman–Halton exact testing likewise confirmed this result (exact *p* < 0.001). Cramér’s V was 0.292. ER = Engagement Rate ([likes + comments]/followers × 100). Correlation strength was interpreted using conventional benchmarks: |r| < 0.20 = negligible/weak; 0.20–0.39 = weak-to-moderate; 0.40–0.59 = moderate; 0.60–0.79 = moderate-to-strong; ≥0.80 = strong [30]. Intraclass correlation coefficients (ICCs) for inter-rater reliability: Descriptive Coverage Index ICC = 0.979 (95% CI: 0.963–0.988); Modified Treatment-Information Reliability score ICC = 0.988 (95% CI: 0.979–0.993) (both *p* < 0.001; [28]).

**Table 6:** Engagement Rate by Descriptive Coverage Index and Modified Treatment-Information Reliability score category.

Quality Category	*n*	Median ER (%)	IQR	H (df)	*p*	Post Hoc (Bonferroni)
Descriptive Coverage Index category						
Low (0–2 points)	85	1.54	3.73	3.977 (2)	0.137	Not significant
Moderate (3–5 points)	340	1.05	3.37			
High (≥6 points)	75	1.91	4.38			
Modified Treatment-Information Reliability score category						
Low (0–2 points)	200	0.88	2.55	8.842 (2)	0.012	High > Low (adj. *p* = 0.033)
Moderate (3–5 points)	257	1.28	3.79			
High (≥6 points)	43	1.85	4.61			

ER = Engagement Rate (%). Kruskal–Wallis test; H statistics represent omnibus comparisons across all three quality categories (Low, Moderate, High) and are shown in the Low-category row for formatting purposes. Bonferroni-corrected post hoc pairwise comparison was applied for the Modified Treatment-Information Reliability score category following a significant omnibus result. Descriptive Coverage Index categories showed no significant difference in ER (H(2) = 3.977, *p* = 0.137). Modified Treatment-Information Reliability score categories showed a significant difference (H(2) = 8.842, *p* = 0.012); post hoc pairwise comparison: High > Low (Bonferroni-adjusted *p* = 0.033); other pairwise comparisons were not significant. Interpretive note: Engagement Rate (ER) served both as the criterion determining inclusion in the Top Posts sampling frame and as the outcome variable in this analysis. This structural circularity precludes causal inference regarding the quality–engagement relationship; findings should be interpreted as reflecting the internal composition of high-visibility posts within the platform’s engagement-based curation, not as evidence of a generalizable association between information quality and audience engagement.

## Data Availability

De-identified raw data underlying the reported analyses are provided as Supplementary Data S1 (Microsoft Excel format). This file contains only post-level numerical scores and classification variables; it does not include usernames, profile links, captions, screenshots, or any other information that could facilitate the identification of individual account holders, in accordance with the privacy and anonymity principles described in Section 2.

## Supplementary Materials

The following supporting information can be downloaded at https://www.mdpi.com/article/10.3390/healthcare14101376/s1: Supplementary Table S1.Descriptive Coverage Index: evaluation items used to assess content scope (maximum score: 8). Supplementary Table S2. Modified Treatment-Information Reliability (MTIR) Score: evaluation items used to assess information reliability (maximum score: 8). Supplementary Table S3. Decision rulebook for post classification (poster source, posting purpose, whitening approach, and post format). Supplementary Table S4. STROBE checklist for cross-sectional studies—completed for the present study. Supplementary Table S5. Supplementary analyses: ordinal logistic regression and approach-stratified Kruskal–Wallis tests for Modified Treatment-Information Reliability Score by poster source. Supplementary Table S6. Item-level frequencies of the Descriptive Coverage Index (DCI) across evaluated Instagram posts (N = 500). Supplementary Table S7. Item-level frequencies of the Modified Treatment-Information Reliability Score (MTIR) across evaluated Instagram posts (N = 500). Supplementary Data S1. De-identified raw dataset (Microsoft Excel format).

## Abbreviations

The following abbreviations are used in this manuscript:

CI	Confidence Interval
DCI	Descriptive Coverage Index
DIY	Do-It-Yourself
ER	Engagement Rate
ICC	Intraclass Correlation Coefficient
IQR	Interquartile Range
IBM	International Business Machines
K-S	Kolmogorov–Smirnov
MTIR	Modified Treatment-Information Reliability Score
OR	Odds Ratio
OTC	Over-the-Counter
SD	Standard Deviation
SPSS	Statistical Package for the Social Sciences
STROBE	Strengthening the Reporting of Observational Studies in Epidemiology
S-W	Shapiro–Wilk
USA	United States of America
