# Supplementary material for: Information Quality, Content Scope, and Audience Engagement of Popular Turkish Instagram Posts on Tooth Whitening: A Cross-Sectional Content Analysis
Source: Healthcare (Basel). 2026 May 18;14(10):1376. doi: 10.3390/healthcare14101376 (PMC13205948; doi:10.3390/healthcare14101376)
Supplement: Supplementary file 1 [file healthcare-14-01376-s001.zip › healthcare-4257585-supplementary.pdf]

# Supplementary Material

## Supplementary evaluation instruments for content scope and information reliability assessment

Both instruments were scored using a binary coding system: 1 point was assigned when the criterion was present in the post, and 0 points when it was absent. The maximum possible score for each instrument was 8. For the Descriptive Coverage Index, the items were adapted from Buldur et al. (2023) [1], whose original instrument employed a binary scoring system from the outset, awarding 1 point for each of the 8 content parameters present in a post (maximum score: 8). The present study adopted this scoring format without modification. For the Modified Treatment-Information Reliability (MTIR) Score, items were adapted from the second and third sections of the original DISCERN instrument [2], covering treatment-specific information quality and overall reliability. The first section of the original DISCERN instrument, which assesses whether a source provides reliable references, is current, and discloses its aims, was deliberately excluded because these criteria are structurally incompatible with social media posts, which rarely cite primary sources, do not display publication dates in a consistent manner, and are not formatted as patient information documents. The items retained from Sections 2 and 3 were considered more applicable to evaluating the clinical information content of short-form visual media. A binary coding approach was preferred over graded scoring for both instruments to reduce interpretive ambiguity and improve coding consistency.

**Table S1.** Descriptive Coverage Index: evaluation items used to assess content scope (maximum score: 8)

| No. | Domain | Evaluation criterion |
|---|---|---|
| 1 | **Definition** | Does the post describe what tooth whitening is and how the treatment works? |
| 2 | **Indications** | Does the post describe the conditions or situations for which whitening is appropriate? |
| 3 | **Contraindications** | Does the post mention conditions in which whitening is not recommended or should be avoided? |
| 4 | **Advantages** | Does the post describe the benefits or expected outcomes of whitening? |
| 5 | **Procedures involved** | Does the post explain the procedural steps or application method of the whitening treatment? |
| 6 | **Complications** | Does the post mention potential side effects, risks, or complications associated with whitening? |
| 7 | **Treatment durability** | Does the post provide information about the longevity or durability of whitening results? |
| 8 | **Professional recommendation** | Does the post advise professional consultation or emphasize dentist supervision when appropriate? |

Adapted from Buldur et al. (2023) [1]; the original instrument employed a binary scoring system (0/1 per criterion; maximum score: 8), which was adopted without modification in the present study. Binary coding: 1 = criterion present; 0 = criterion absent.

**Table S2.** Modified Treatment-Information Reliability (MTIR) Score: evaluation items used to assess information reliability (maximum score: 8)

| No. | Domain | Evaluation criterion |
|---|---|---|
| 1 | **Treatment mechanism** | Does it describe how each treatment works? |
| 2 | **Treatment benefits** | Does it describe the benefits of each treatment? |
| 3 | **Treatment risks** | Does it describe the risks of each treatment? |
| 4 | **No-treatment option*** | Does it describe what may occur if no whitening treatment is used? |
| 5 | **Quality of life** | Does it describe how treatment choices affect overall quality of life? |
| 6 | **Treatment alternatives** | Is it clear that there may be more than one possible treatment choice? |
| 7 | **Areas of uncertainty** | Does it refer to areas of uncertainty or gaps in current knowledge? |
| 8 | **Overall informational quality** | Does the post demonstrate adequate overall informational coherence, clarity, internal consistency, and relevance to its stated topic? |

Adapted from Charnock et al. (1999) [2], Sections 2 and 3. Section 1 items (source reliability, currency, and disclosure of aims) were excluded because they were structurally incompatible with the social media post format. Binary coding: 1 = criterion present; 0 = criterion absent. For the overall quality item, a binary score was assigned when the post met a predefined threshold of overall informational coherence, including clarity, internal consistency, and the presence of sufficiently complete and relevant information for its stated topic. *Item 4 (No-treatment option) was retained from the original DISCERN Section 2 for scoring completeness; however, in the context of cosmetic tooth whitening social media posts, this criterion is expected to have limited discriminatory power, as the consequences of forgoing whitening are rarely addressed in such content.

**Table S3.** Decision rulebook for post classification (poster source, posting purpose, whitening approach, and post format)

This rulebook defines the decision criteria applied by both evaluators during post-classification. In cases of ambiguity, classification was determined by consensus between the two evaluators; where uncertainty persisted, a third senior researcher was consulted. All four classification variables were applied to each included post prior to content scoring.

## Section A. Poster Source (3 categories)

| Category | Classification criteria |
|---|---|
| **Dentist/clinic account** | Profile explicitly states a professional clinical title (e.g., "dentist," "diş hekimi," "periodontist") and/or shows clear institutional affiliation (dental clinic, hospital dental department, dental school); or the account is operated under the name or branding of a dental practice or clinic. Applies regardless of whether posts are educational or promotional. |
| **Independent user** | An individual personal account with no stated professional dental qualification, no institutional affiliation, and no commercial branding. Typically shares personal experience, testimonials, or lifestyle content. Influencer accounts without dental credentials are coded here regardless of follower count. |
| **Brand/commercial account** | Account operated by a company, manufacturer, or retailer that produces or sells dental/cosmetic products or consumer brands with a whitening-related product line. Profile explicitly identifies a commercial entity or brand name without professional dental credentials. |

**Section B. Posting Purpose (3 categories)**

| Category | Classification criteria and decision rules |
|---|---|
| **Educational** | Dominant intent is to inform, explain, or raise awareness about tooth whitening. Characterized by explanatory text, procedural descriptions, clinical information, FAQ formats, myth-busting content, or safety guidance. A promotional call-to-action may be incidentally present but must not constitute the dominant message. Decision rule: if the primary frame is "here is information you should know," code as Educational. |
| **Marketing** | Dominant intent is to promote a service, product, or practice. Characterized by before-and-after treatment images as promotional material, explicit calls-to-action ("book now," "contact us," "get 20% off"), product advertisements, or clinic promotional content. Decision rule: if the primary frame is "use our service/product," code as Marketing. Before-and-after photos without a primary informational narrative are coded as Marketing by default. |
| **Experience-based** | Dominant intent is to share personal experience. Characterized by first-person narration, testimonial content, patient accounts, vlog-style documentation, or personal product reviews. Decision rule: if framed as "here is what happened to me / what I tried," code as Experience-based. Posts with affiliate links are coded here only if the experiential narrative is clearly dominant; otherwise, code as Marketing. |

Posts intended solely for entertainment purposes were excluded prior to classification. For this criterion, 'entertainment-only' was operationally defined as posts whose primary communicative intent was humor, personal performance, or non-dental lifestyle content, with no discernible tooth-whitening information component. Disagreements between evaluators regarding this classification were resolved by consensus discussion.

**Section C. Whitening Approach (5 categories)**

| Category | Classification criteria and examples |
|---|---|
| **In-office whitening** | Post primarily addresses professionally administered whitening performed in a dental clinic. Examples include bleaching with high-concentration hydrogen peroxide or carbamide peroxide gels (with or without light activation), power bleaching (e.g., Zoom, Beyond), and clinician-supervised take-home trays. Posts describing clinician-supervised take-home tray whitening are also classified here, as the defining clinical characteristic of this approach, professional examination and custom tray fabrication, occurs within the clinical setting, regardless of where the agent is applied |
| **OTC products** | Post primarily addresses commercially manufactured whitening products for self-use without professional supervision. Examples include whitening strips, whitening toothpastes, whitening pens, whitening mouthwashes, and OTC whitening kits. Must be a named commercial item; general references to "whitening products" without specification are coded as Unspecified/other. |
| **DIY/natural home remedies** | Post primarily addresses home-made or natural whitening methods using household or natural ingredients. Examples include baking soda, activated charcoal, oil pulling, lemon or strawberry pulp, and turmeric. Distinguishing feature: no manufactured product brand; preparation is described as self-initiated using non-commercial ingredients. |
| **Multiple methods** | Post comparably addresses two or more whitening approach categories. Decision rule: code as Multiple methods only when no single category accounts for more than approximately 60% of content; if one approach is clearly predominant, code to that category instead. |
| **Unspecified/other** | Post relates to tooth whitening but does not sufficiently specify a particular approach. Examples include general aesthetic result posts without method detail, or posts where the approach cannot be determined. Used as a residual category only when none of the above apply. |

**Section D. Post Format (3 categories)**

| Category | Definition |
|---|---|
| Photo | A single static image post, as classified by the Instagram platform interface. Identified by the absence of a video playback indicator and the absence of a multi-panel swipe indicator in the post display. |
| Reels | A short-form vertical video post, as classified by the Instagram platform interface. Identified by the Reels-specific playback interface (vertical video with audio, sharing, and reaction controls). |
| Carousel | A multi-panel post consisting of two or more images or videos displayed as a swipeable sequence, as classified by the Instagram platform interface. Identified by the multi-panel swipe indicator (dot navigation or arrow icon) visible in the post display. |

**Table S4**. STROBE checklist for cross-sectional studies — completed for the present study

| Item | Section / Topic | Item requirement (STROBE cross-sectional) | Reported on page/section |
|---|---|---|---|
| 1 | **Title and abstract** | (a) Indicate the study's design in the title or abstract using a commonly used term. (b) Provide in the abstract an informative and balanced summary of what was done and what was found. | Title ("Cross-Sectional Content Analysis"); Abstract section |
| 2 | **Background/rationale** | Explain the scientific background and rationale for the investigation being reported. | Introduction section |
| 3 | **Objectives** | State specific objectives, including any pre-specified hypotheses. | Introduction (final paragraph): RQ1 stated as primary aim; RQ2 stated as secondary, exploratory question; Abstract – Purpose |
| 4 | **Study design** | Present key elements of the study design early in the paper. | Methods – Study Design; Abstract – Materials and Methods |
| 5 | **Setting** | Describe the setting, locations, and relevant dates, including periods of recruitment, exposure, follow-up, and data collection. | Methods – Data Source and Sample Selection (Instagram, #dişbeyazlatma, 1–5 November 2025) |
| 6 | **Participants** | Give the eligibility criteria, and the sources and methods of selection of participants. For cross-sectional studies: describe the study unit and selection procedures. Give reasons for any exclusions. | Methods – Data Source and Sample Selection (inclusion/exclusion criteria; n = 500) |
| 7 | **Variables** | Clearly define all outcomes, exposures, predictors, potential confounders, and effect modifiers. Give diagnostic criteria, if applicable. | Methods – Data Recording and Classification; Content Evaluation; Supplementary Tables S1–S3 |
| 8 | **Data sources/ measurement** | For each variable of interest, give sources of data and details of the methods of assessment. Describe the comparability of assessment methods if there is more than one group. | Methods – Content Evaluation; Calibration and Inter-Rater Reliability; Supplementary Tables S1–S2 |

| Item | Section / Topic | Item requirement (STROBE cross-sectional) | Reported on page/section |
|---|---|---|---|
| 9 | **Bias** | Describe any efforts to address potential sources of bias. | Methods – Data Source (new account; algorithmic bias mitigation); Calibration and Inter-Rater Reliability; Discussion – Limitations |
| 10 | **Study size** | Explain how the study size was arrived at. | Methods – Data Source and Sample Selection (n = 500; pragmatic determination; post-hoc power noted) |
| 11 | **Quantitative variables** | Explain how quantitative variables were handled in the analyses. If applicable, describe which groupings were chosen and why. | Methods – Content Evaluation (binary scoring, 0–2/3–5/≥6 categories); Statistical Analysis |
| 12 | **Statistical methods** | Describe all statistical methods, including those used to control for confounding. Describe any methods used to examine subgroups and interactions. Explain how missing data were addressed. Describe any sensitivity analyses. | Methods – Statistical Analysis (Kruskal-Wallis with $\eta^2$ effect size [3], Bonferroni, chi-square/Fisher, Spearman; ordinal logistic regression; SPSS v26.0; no missing data reported). No missing data were present in the analytic dataset, as all included posts were fully accessible, and all required variables were recorded at the time of archiving. |
| 13 | **Participants (flow)** | Report the number of individuals at each stage of the study. Consider using a flow diagram. | Methods – Data Source and Sample Selection (flow diagram provided as Figure 1; total screened, per-criterion exclusion counts, and final n = 500 reported therein); Results – Descriptive Statistics (n = 500; 0 missing) |
| 14 | **Descriptive data** | Give characteristics of study participants and summary information on exposures and potential confounders. | Results – Descriptive Statistics; Table 1 |
| 15 | **Outcome data** | Report numbers of outcome events or summary measures. | Results – Content and Information Quality Scores; Tables 2–6 |
| 16 | **Main results** | Give unadjusted estimates and, if applicable, confounder-adjusted estimates and their precision. Report the category boundaries for continuous variables that were categorized. If relevant, consider translating relative risk estimates into absolute risk for a meaningful time period. | Results sections: Tables 3–6 (H statistics, p values, $\eta^2$, Spearman r) |
| 17 | **Other analyses** | Report other analyses done (subgroup analyses, interaction analyses, sensitivity analyses). | Results – Supplementary analyses: ordinal logistic regression (poster source × whitening approach; Supplementary Table S5); approach-stratified Kruskal- |

| Item | Section / Topic | Item requirement (STROBE cross-sectional) | Reported on page/section |
|---|---|---|---|
| | | | Wallis tests (Supplementary Table S5); Engagement Rate by quality-score category (Table 6) |
| 18 | **Key results** | Summarize key results with reference to study objectives. | Discussion (first two paragraphs); Conclusion |
| 19 | **Limitations** | Discuss limitations of the study, taking into account sources of potential bias or imprecision. Discuss both direction and magnitude of any potential bias. | Discussion – Limitations paragraph (8 limitations explicitly listed) |
| 20 | **Interpretation** | Give a cautious overall interpretation of results considering objectives, limitations, multiplicity of analyses, results from similar studies, and other relevant evidence. | Discussion (throughout); Conclusion |
| 21 | **Generalizability** | Discuss the generalizability (external validity) of the study results. | Conclusion: 'Given the observational design and hashtag-based sampling frame, generalization beyond the conditions of this study is not warranted'; external validity further discussed in Discussion – Limitations (single hashtag, Top Posts algorithm, five-day data collection window, Turkish-language context) |

Adapted from von Elm et al. (2007) [4]. STROBE = Strengthening the Reporting of Observational Studies in Epidemiology. Items are numbered according to the original STROBE cross-sectional checklist. "Reported on page/section" column refers to the location in the manuscript where each item is addressed. N/A = not applicable to this study design.

**Table S5.** Supplementary analyses: ordinal logistic regression and approach-stratified Kruskal-Wallis tests for Modified Treatment-Information Reliability Score by poster source

These analyses were conducted to assess whether the source-level advantage in the Modified Treatment-Information Reliability Score persisted after controlling for the whitening approach. Section A presents the ordinal logistic regression model (logit link; proportional odds). Section B presents approach-stratified nonparametric comparisons of the Modified Treatment-Information Reliability Score and the Descriptive Coverage Index across poster source categories. Reference categories: poster source = brand/commercial accounts; whitening approach = unspecified/other. OR = odds ratio; CI = confidence interval; n.s. = not significant (Bonferroni-adjusted $p \geq .05$); N/A = not applicable (insufficient group size or single-group stratum).

**Section A. Ordinal logistic regression: Modified Treatment-Information Reliability Score category as outcome**

Model fit: $\chi^2(6) = 132.178$, $p < .001$; Nagelkerke $R^2 = .276$; Pearson goodness-of-fit $p = .220$; Deviance $p = .043^a$; Test of Parallel Lines: $\chi^2(6) = 9.449$, $p = .150$ (proportional odds assumption met). N = 500; zero missing.

| Variable | B (Estimate) | Wald | p | OR | 95% CI |
|---|---|---|---|---|---|
| **Poster source (ref = brand/commercial)** | | | | | |
| Dentist/clinic | 1.265 | 6.417 | **.011** | $3.54^b$ | 1.33–9.42 |

| Variable | B (Estimate) | Wald | p | OR | 95% CI |
|---|---|---|---|---|---|
| Independent user | 0.699 | 2.607 | .106 | 2.01 | 0.86–4.70 |
| **Whitening approach (ref = unspecified/other)** | | | | | |
| In-office | 1.656 | 38.071 | **<.001** | 5.24 | 3.09–8.86 |
| OTC | 1.328 | 7.328 | **.007** | 3.77 | 1.44–9.87 |
| DIY | 1.080 | 3.897 | **.048** | 2.94 | 1.01–8.59 |
| Multiple methods | 3.681 | 91.316 | **<.001** | 39.70 | 18.56–84.79 |

**Section B. Approach-stratified non-parametric tests: Modified Treatment-Information Reliability (MTIR) Score and** Descriptive Coverage Index(DCI) **by poster source**

| Whitening approach stratum | n | Test | MTIR H | MTIR p | Content p | Source groups compared | Key post-hoc finding (MTIR) |
|---|---|---|---|---|---|---|---|
| In-office | 222 | Kruskal-Wallis | 5.675 | .059 | .921 | DH/K, IU, B (n = 212, 6, 4) | No significant pairwise differences (all adj. p ≥ .05) |
| OTC | 85 | Kruskal-Wallis | 7.172 | .028 | .691 | DH/K, IU, B (n = 9, 31, 45) | DH/K > B (adj. p = .022); DH/K vs. IU, n.s. (adj. p = .112) |
| DIY | 38 | Mann-Whitney U | — | .003 (exact) | .045 (exact) | DH/K (n = 5) vs IU (n = 33) only | Statistically significant (exact p = .003), but DH/K n = 5 is insufficient for substantive interpretation; result is directionally consistent with overall source effect only |
| Multiple methods | 66 | Mann-Whitney U | — | .411 | .630 | DH/K (n = 64) vs IU (n = 2) only | Not interpretable (n = 2 for IU; insufficient statistical power) |
| Unspecified/other | 89 | Not tested (single-source stratum: all posts from dentist/clinic accounts; no between-group comparison possible) | | | | | |

DH/K = dentist/clinic accounts; IU = independent users; B = brand/commercial accounts. Significant p values (< .05) are shown in bold. adj. p = Bonferroni-corrected pairwise p value. The unspecified/other stratum was used as the reference category in the ordinal logistic regression model. OR values were computed as exp(B).

[a] Note A: The statistical significance of the Deviance goodness-of-fit statistic (p = .043) is attributable to sparse cells arising from the strong correlation between poster source and whitening approach, rather than reflecting inadequate overall model fit. The Pearson goodness-of-fit statistic (p = .220) confirms acceptable model fit and should be regarded as the primary fit index in this context.

[b] Note B: OR values derived from this model, such as OR = 3.54 for dentist/clinic accounts, should be interpreted as directional signals favoring dentist/clinic accounts rather than as precise quantitative estimates, given the sparse cell counts in several predictor strata.

**Table S6. Item-level frequencies of the Descriptive Coverage Index (DCI) across evaluated Instagram posts (N = 500)**

| No. | Content Domain | Evaluation Criterion (abbreviated) | Frequency (n) | Percentage (%) |
|---|---|---|---|---|
| 1 | **Definition** | Does the post describe what tooth whitening is and how it works? | 256 | 51.2% |
| 2 | **Indications** | Does the post describe conditions for which whitening is appropriate? | 437 | 87.4% |
| 3 | **Contraindications** | Does the post mention conditions in which whitening is not recommended? | 91 | 18.2% |
| 4 | **Advantages** | Does the post describe the benefits or expected outcomes of whitening? | 442 | 88.4% |
| 5 | **Procedures involved** | Does the post explain the procedural steps or application method? | 390 | 78.0% |
| 6 | **Complications** | Does the post mention potential side effects, risks, or complications? | 215 | 43.0% |
| 7 | **Treatment durability** | Does the post provide information about the longevity of whitening results? | 110 | 22.0% |
| 8 | **Professional recommendation** | Does the post advise professional consultation or emphasize dentist supervision when appropriate? | 23 | 4.6% |

**Note.** Binary coding: 1 = criterion present; 0 = criterion absent. Items adapted from Buldur et al. (2023)[1]. Items are listed in their original instrument order. Percentages reflect the proportion of posts (N = 500) in which each criterion was coded as present.

**Table S7. Item-level frequencies of the Modified Treatment-Information Reliability Score (MTIR) across evaluated Instagram posts (N = 500)**

| No. | Reliability Domain | Evaluation Criterion (abbreviated) | Frequency (n) | Percentage (%) |
|---|---|---|---|---|
| 1 | **Treatment mechanism** | Does it describe how each whitening treatment works? | 230 | 46.0% |
| 2 | **Treatment benefits** | Does it describe the benefits of each whitening treatment? | 427 | 85.4% |
| 3 | **Treatment risks** | Does it describe the risks of each whitening treatment? | 107 | 21.4% |
| 4 | **No-treatment option** | Does it describe what may occur if no whitening treatment is used? | 224 | 44.8% |
| 5 | **Quality of life** | Does it describe how treatment choices affect overall quality of life? | 266 | 53.2% |
| 6 | **Treatment alternatives** | Is it clear that there may be more than one possible whitening treatment? | 89 | 17.8% |
| 7 | **Areas of uncertainty** | Does it refer to areas of uncertainty or gaps in current knowledge? | 138 | 27.6% |
| 8 | **Overall quality** | Does the post demonstrate adequate overall informational coherence and clarity? | 97 | 19.4% |

Note. Binary coding: 1 = criterion present; 0 = criterion absent. Items adapted from Charnock et al. (1999) [2], Sections 2 and 3. Section 1 items (source reliability, currency, and disclosure of aims) were excluded because they were structurally incompatible with the social media post format. Item 4 (No-treatment option) was retained for scoring completeness; its discriminatory power in cosmetic whitening content is limited, given the low clinical consequence of forgoing whitening. Item 8 (Overall quality) was scored using predefined thresholds for informational coherence, clarity, and internal consistency. Percentages reflect the proportion of posts (N = 500) in which each criterion was coded as present.

## References


1. Buldur, M.; Misilli, T.; Ayan, G. Analyzing Content and Information Quality of Instagram® Posts About #teethwhitening. *Cumhuriyet Dental Journal* **2023**, *26*, 268–275, doi:10.7126/cumudj.1262248.
2. Charnock, D.; Shepperd, S.; Needham, G.; Gann, R. DISCERN: An instrument for judging the quality of written consumer health information on treatment choices. *Journal of Epidemiology and Community Health* **1999**, *53*, 105–111, doi:10.1136/jech.53.2.105.
3. Tomczak, M.; Tomczak, E. The need to report effect size estimates revisited. An overview of some recommended measures of effect size. *Trends in Sport Sciences* **2014**, *1*, 19–25.
4. von Elm, E.; Altman, D.G.; Egger, M.; Pocock, S.J.; Gøtzsche, P.C.; Vandenbroucke, J.P. The Strengthening the Reporting of Observational Studies in Epidemiology (STROBE) statement: Guidelines for reporting observational studies. *Lancet* **2007**, *370*, 1453–1457, doi:10.1016/S0140-6736(07)61602-X.
